# Evaluation of *Plasmodium falciparum* MSP10 and its development as a serological tool for the Peruvian Amazon region

**DOI:** 10.1186/s12936-019-2959-8

**Published:** 2019-09-23

**Authors:** Jorge Bendezu, Elizabeth Villasis, Sandra Morales Ruiz, Katherine Garro, Berónica Infante, Renzo Gutierrez-Loli, Pamela Rodríguez, Manolo Fernández-Díaz, Dionicia Gamboa, Katherine Torres

**Affiliations:** 1 Laboratorios de Investigación y Desarrollo, FARVET, Carretera Panamericana Sur No 766 km 198.5, Chincha Alta, Ica, Peru; 2 Laboratorios de Investigación y Desarrollo “Abraham Vaisberg Wolach, Facultad de Ciencias y Filosofía, Universidad Peruana Cayetano Heredia, Lima, Peru; 3 Instituto de Medicina Tropical Alexander von Humboldt-Universidad Peruana Cayetano Heredia, Av. Honorio Delgado 430, San Martín de Porres, Lima, Peru

**Keywords:** *Plasmodium falciparum*, PfMSP10, Monoclonal antibodies, Peptides

## Abstract

**Background:**

Different antigens are needed to characterize *Plasmodium falciparum* infection in terms of seroreactivity and targets for invasion inhibition, in order to guide and identify the proper use of such proteins as tools for the development of serological markers and/or as vaccine candidates.

**Methods:**

IgG responses in 84 serum samples from individuals with *P. falciparum* infection [classified as symptomatic (Sym) or asymptomatic (Asym)], or acute *Plasmodium vivax* infection, from the Peruvian Amazon region, were evaluated by enzyme-linked immunosorbent assays specific for a baculovirus-produced recombinant protein *P. falciparum* Merozoite Surface Protein 10 (rMSP10) and for non-EGF region selected peptides of PfMSP10 selected by a bioinformatics tool (PfMSP10-1, PfMSP10-2 and PfMSP10-3). Monoclonal antibodies against the selected peptides were evaluated by western blotting, confocal microscopy and inhibition invasion assays.

**Results:**

Seroreactivity analysis of the *P. falciparum* Sym- and Asym-infected individuals against rMSP10 showed a higher response as compared to the individuals with *P. vivax* acute infection. IgG responses against peptide PfMSP10-1 were weak. Interestingly high IgG response was found against peptide PfMSP10-2 and the combination of peptides PfMSP10-1 + PfMSP10-2. Monoclonal antibodies were capable of detecting native PfMSP10 on purified schizonts by western blot and confocal microscopy. A low percentage of inhibition of merozoite invasion of erythrocytes in vitro was observed when the monoclonal antibodies were compared with the control antibody against AMA-1 antigen. Further studies are needed to evaluate the role of PfMSP10 in the merozoite invasion.

**Conclusions:**

The rMSP10 and the PfMSP10-2 peptide synthesized for this study may be useful antigens for evaluation of *P. falciparum* malaria exposure in Sym and Asym individuals from the Peruvian Amazon region. Moreover, these antigens can be used for further investigation of the role of this protein in other malaria-endemic areas.

## Background

In 2017, ∼219 million cases of malaria were registered worldwide, causing 435,000 deaths mainly due to *Plasmodium falciparum* [1]. In Peru, 44,406 cases of malaria were reported in 2018 and 20% of them were due to *P. falciparum* [2]. Thus, an effective vaccine against falciparum malaria remains a major goal for global public health [3]. The use of the invasion proteins from the merozoite specific-stage could be attractive for malaria vaccine development because this stage is exposed directly to the humoral immune system of the host. Likewise, antibodies against invasion proteins block the merozoite-erythrocyte interaction, decreasing the parasitaemia as well as the clinical symptoms of malaria-infected individuals and therefore transmission [4].

Several studies have also suggested the potential application of *P. falciparum* merozoite proteins in sero-epidemiological surveillance [4, 5]. Moreover, studies in the Peruvian Amazon region have revealed the presence of a large number of non-febrile or asymptomatic infections that can occur over short time periods. Additionally, individuals with asymptomatic infections are able to develop and maintain IgG responses against the *P. falciparum* merozoite for up to a 6-month period, in contrast to the response seen in high transmission areas such as Africa [5–7]. Recently, *P. falciparum* Merozoite Surface Protein 10 (PfMSP10) was demonstrated to have high reactivity to the serum samples from symptomatic (Sym) and asymptomatic (Asym) *P. falciparum*-infected individuals from the Peruvian Amazon region, on platforms as the protein-array and enzyme-linked immunosorbent assay (ELISA) [6, 8].

PfMSP10 is found in the surface and the apical region of the merozoite. It possesses two Epidermal Growth Factor (EGF) domains, and its C-terminus is anchored in the erythrocyte membrane [9]. The function of this protein remains unknown; however, it is believed to be associated with growth promotion and protein–protein recognition in merozoites and gametocytes [9].

Antibodies against the N-terminal region of this protein only recognize an 80 kDa band, whereas antibodies against its C-terminal region have been reported to react with 80-kDa and 36-kDa bands in lysed *P. falciparum* parasites, suggesting that the PfMSP10 protein is subjected to processing in a manner similar to that of PfMSP1 and PfMSP8, but with a smaller number of cleavage events [9, 10]. Isolated human antibodies can recognize conformational epitopes of the EGF-like domain of PfMSP10 and inhibit the process of invasion of merozoites into the erythrocyte under in vitro conditions [11]. Therefore, the PfMSP10 protein and its EGF domain could be a valuable antigen, for the design of malaria vaccines. However, the functions of other non-EGF domains of PfMSP10 have not yet been fully explored [9, 10].

Recombinant antigens can be produced in different expression systems (*Escherichia coli*, yeast and mammalian cells) and their use has improved the procedures of evaluation of IgG responses to malaria antigens in large-scale studies [5, 8]; besides, synthetic peptides have been used for the development of tools for sero-epidemiological surveys [12, 13], and the selection of these peptides is facilitated by the use of bio-informatics tools; furthermore, these peptides can be employed for antibody generation [14].

The aim of this work was to explore the utility PfMSP10 as a surveillance tool. For this purpose, a recombinant PfMSP10 protein was produced in an eukaryotic low-demanding system as baculovirus and evaluated the seroreactivity against this protein and synthetic peptides in terms of IgG responses on sera from individuals living in the Peruvian Amazon. Within this study, PfMSP10 was confirmed as a potential serological marker for this malaria-endemic region and evaluated the role of antibodies against selected peptides from non-EGF domains of PfMSP10, during merozoite invasion.

## Methods

### Baculovirus vector construction using *pfmsp10* gene

The full-length *pfmsp10* gene without the intron was obtained from PlasmoDB using a Gene ID (PF3D7_0620400) [15]. Then, two segments (nucleotide positions (nt): (1) from 1 to 78 nt and (2) from 1519 to 1575 nt) were excluded owing to the presence of a signal peptide and a potential transmembrane domain, respectively. Then, the *pfmsp10* gene (from 79 to 1518 nt) was codon-optimized for expression in insect cells (Sf9) and an 8×-His tag was placed on the carboxyl side of the protein (Additional file 1: Figure S1), and synthesized in pUC57 vector (GenScript Laboratories, Piscataway, NJ, USA). First, construction of the baculovirus pFastBac-*pfmsp10* vector was carried out using the following primers: (1) F1 primer [sequence: 5′-ATTGGATCCATT**ATG**CACGTGGATGACATCAAG-3′; with a BamHI cloning site (letters underlined)] and (2) R1 primer [sequence: 5′-TTAGAATTCTTA**TTA**ATGATGATGATGATGATGATGCTTG-3′; with an EcoRI cloning site (letters underlined)]; the start and stop codons are shown in bold in the F1 and R1 primer sequences, respectively. Second, *pfmsp10* was cloned into the pFastBacTM dual vector (Invitrogen, Carlsbad, CA, USA) between BamHI and EcoRI sites from pUC57 vector with *pfmsp10* gene using F1 and R1 primers (Additional file 1: Figure S2). Finally, the reading frame was verified by a sequencing service (Macrogen, Seoul, Korea).

### Preparation of a recombinant baculovirus and transfection of insect cells with the pFastBac-*pfmsp10* vector

Recombinant baculovirus was produced using the pFastBac-*pfmsp10* vector, DH10Bac competent cells (Invitrogen) and a *Spodoptera frugiperda* (Sf9) cell line (Invitrogen), following published instructions [16]. The first generation of the recombinant baculovirus was designated as P0, and then baculoviruses were passaged two times to obtain a new generation called P2.

### Expression of recombinant *Plasmodium falciparum* Merozoite Surface Protein 10 (rMSP10)

Two hundred-fifty mL flasks were prepared and 0.8 × 10^6^ Sf9-insect cells were seeded in a volume of 100 mL. The flasks were maintained under constant agitation (100 × g/27 °C) overnight, and on the following day, using the P2 generation, the Sf9-insect cell flasks were infected at a multiplicity of infection (MOI) of 20 and the cells were harvested after 48 h. The harvested cells were lysed with lysis buffer consisting of 50 mM Tris–HCl, 5 mM EDTA, 300 mM NaCl, 10%[v/v] of dimethyl sulfoxide (Sigma Aldrich, St, Louis, MO, USA) and 0.6% w/v CHAPS (Sigma Aldrich). SDS-PAGE and western blot assays were performed using lysate resuspended in 4× single buffer (40% [v/v] of glycerol, 240 mM Tris/HCl at pH 6.8, 8% of SDS, 0.04% of bromophenol blue and 5% [v/v] of beta-mercaptoethanol). The sample was then placed in a boiling water bath for 5 min and centrifuged at 10,000×*g* for 5 min, and 10 µL of the supernatant was loaded onto a gel for SDS-PAGE. The samples were run for 120 min at 0.01A for each gel. The proteins were transferred from the gel to a nitrocellulose membrane using an e-blot system (GenScript, Piscataway, NJ, USA) following the manufacturer’s instructions. Western blotting was performed with a pre-prepared membrane as described. The membranes were immersed in TTBS-BSA blocking buffer (50 mM Tris, 150 mM NaCl, 0.05% of Tween 20, pH 7.6, with 1% of BSA of [w/v]) for 1 h with agitation. The membrane was then placed in a SNAPid 2.0 protein detection system (EMD Millipore, Billerica, MA, USA) following the manufacturer’s instructions until the membranes were ready for development, using an anti-His antibody (0.001 mg/mL) (Genscript) and TTBS as a washing buffer. The membranes were then developed using SIGMAFAST DAB with Metal Enhancer tablets (Sigma Aldrich). The tablets were diluted in 10 mL of TTBS buffer, and 5 mL of this solution was added to each membrane with agitation for a 15-min incubation until the bands developed. The reaction was stopped with distilled water. Molecular markers were included during SDS-PAGE (BioRad, Hercules, CA, USA).

### rMSP10 purification assays

The proteins were purified by affinity chromatography for detection of the histidine tag. For this purpose: 20 million infected insect cells were lysed per 1 mL of lysis buffer (1% of Tween-20, 10% [v/v] of DMS0, 100 mM Tris–HCl and 250 mM NaCl) in the presence of phenylmethylsulfonyl fluoride (PMSF) (Sigma Aldrich), followed by incubation with agitation at 4 °C overnight. The next day, the lysates were centrifuged at 10,000×*g* for 10 min at 4 °C, and the supernatant was then removed and passed through a 0.22-µm membrane. The supernatant was next loaded onto an immobilized metal affinity chromatography column (IMAC) (BioRad, Hercules, CA, USA) using BioLogic LP System (BioRad), according to the standard conditions for the purification of soluble proteins in an imidazole gradient (0–500 mM). At the end of this step, 4 mL fractions were collected. All the fractions were concentrated using 100 molecular weight cutoff (MWCO) ultrafiltration membranes (EMD Millipore, Billerica, MA, USA). The concentrated fractions with rMSP10 were quantified by the Bradford method (BioRad) following the manufacturer’s instructions. Aliquots of rMSP10 were stored at − 80 °C for further procedures.

### PfMSP10 peptides

Peptides PfMSP10-1, PfMSP10-2 and PfMSP10-3 were selected within the PfMSP10 protein sequence (GenBank:XP_966190.1) using the Optimum Antigen design tool (GenScript Laboratories). This algorithm considers characteristics as antigenicity, hydrophilicity/hydrophobicity, secondary structure, disordered score, and common sequence motifs, and integrates commercially available algorithms to optimize the design of antigenic peptides. PfMSP10 peptides were selected considering their hits according this bioinformatics tool (Additional file 1: Table S1); then, peptides were synthesized by GenScript Laboratories and employed for further assays.

### Serum samples

A total of 54 serum samples from individuals infected with *P. falciparum* and 30 serum samples from individuals infected with *Plasmodium vivax* were analysed in this study. All serum samples were collected from 17 different communities in the Department of Loreto, Peru, from 2008 to 2016 in previous studies [6, 17].

*Plasmodium falciparum* samples were sub-divided into two groups: (1) 30 individuals with Sym *P. falciparum* infection; and, (2) 24 individuals with Asym *P. falciparum* infection. Sym individuals were enrolled by passive case detection at San Juan de Miraflores Health Center in the District of San Juan (Iquitos City, Capital of the Loreto Department, Peru). Most Asym subjects were identified by active case detection after a malaria diagnostic test was performed in Atalaya and Diamante Azul, small villages located 6–7 km from Iquitos by motorboat in the district of Alto Nanay.

All *Plasmodium * spp. infections were confirmed by light microscopic examination of stained thick and thin peripheral blood smears. The microscopy diagnosis was later confirmed by PCR [18]. Blood samples were collected before all individuals were treated for malaria, following the National Drug Policy Guidelines of the Peruvian Ministry of Health.

### Indirect ELISAs for *Plasmodium falciparum* and *Plasmodium vivax*

The reactivity of serum samples towards peptide rMSP10 or PfMSP10 peptides was assessed by indirect ELISA. This assay was performed in Costar High Binding plates (Sigma Aldrich). Individual wells were coated with the recombinant protein or synthetic peptide, at 5 µg/mL, and blocked with phosphate buffered saline (PBS) containing 0.1% of bovine serum albumin (BSA). Serum samples were diluted 1:100 in 1.5% non-fat milk washing solution (0.15 M Na_2_HPO_4_, 0.15 M NaH_2_PO_4_, 0.44 M NaCl, 0.05% of Tween20, and 0.05% of BSA). IgG was detected using peroxidase-conjugated AffiniPure Goat anti-human-IgG antibody (Jackson Immuno Research, West Grove, PA, USA) diluted 1:2000. Later, the plates were washed and 100 μL/well of 3,3′,5,5′-tetramethylbenzidine (TMB) substrate (BD OptEIA, San Jose, CA, USA) was added. The reaction was stopped using 50 μL/well of 0.25 M HCl, and the absorbance was read at 450 nm on an ELISA iMARK microplate-reader (BioRad). Positive controls included five samples from five *P. falciparum*-infected individuals and a ‘positive pool’ made of three samples. The positive pool control was used for the construction of a standard-curve (1:50, 1:100, 1:400, 1:1600, 1:3200, and 1:12,800) which was built for each experiment, yielding similar results and to allow us defined the correct serum dilution for the further assays (Additional file 1: Figure S3).

### Serum samples and antigens for cross-reactivity controls

*Plasmodium vivax* samples were used as specific-controls for the antigens (*P. falciparum* recombinant protein and synthetic peptides) because *P. falciparum* and *P. vivax* infections coexist in the Peruvian Amazon region. The recombinant protein *P. vivax* MSP1 (PvMSP1), purchased from MyBioSource, Inc (San Diego, CA, USA) served as a control to evaluate cross-species reaction among patients infected with *P. falciparum.* An indirect ELISA was established using PvMSP1 following all the steps describe above.

To test the specificity of the antigens produced in this study, serum samples were evaluated from patients with non-malaria related infections with known presence in the Peruvian Amazon region. The serum from patients with other infections such as toxoplasmosis (Tx; n = 1), leishmaniasis (Ls; n = 1), leptospirosis (Lp; n = 1), dengue (Dn; n = 3), and neurocysticercosis (Nc; n = 1) were selected.

Furthermore, serum samples from two individuals living in endemic areas with negative diagnostic for malaria according to PCR and microscopy and one serum sample from a healthy donor not living in a malaria-endemic area were employed to determinate the cut-off for ELISA assays.

### Monoclonal antibodies (Ab)

Each synthetic peptide was used to produce monoclonal antibodies called AbMSP10-1, AbMSP10-2 and AbMSP10-3 (GenScript Laboratories) (Table 1). A monoclonal antibody against *P. falciparum* Apical Membrane Antigen 1 (abAMA1) from clone N4-1F6 was acquired from MR4/BEI Resources, NIAD, NIH; and used as a control for the invasion inhibition assays (IIA) and confocal microscopy.

**Table 1:** Description of peptides and monoclonal antibodies used in this study

Name of peptide	Peptide sequence	Amino acid position^a^	Region in PfMSP10	Monoclonal antibody (Ab)	Concentration [mg/mL]
PfMSP10-1	NMNNEKNDNKDNKD	Asn50-Asp63	N-terminal	AbMSP10-1	0.50
PfMSP10-2	ERKNQKAPPGEHKP	Glu306-Pro319	Central	AbMSP10-2	1.38
PfMSP10-3	RRTLLKESRDIKNT	Arg368-Thr381	Central	AbMSP10-3	2.45

^a^*P. falciparum* MSP10 protein sequence from strain 3D7. (PlasmoDB: PF3D7_0620400)

### Parasite culture

Asexual parasites from *P. falciparum* strain 3D7 (MR4, Manassas, VA, USA) were maintained in O + human erythrocytes at 5% haematocrit in RPMI-HEPES supplemented with 10% human pool serum AB + , according to standardized methods [19]. Cultures were maintained in synchronized stages by the sorbitol methodology [20]. Parasites were used for evaluation of the monoclonal antibodies by western blotting, confocal microscopy and IIAs.

### Western blot assays

A purified pellet from synchronized schizont stages of the *P. falciparum* 3D7 strain was separated by SDS-PAGE in a 12% polyacrylamide gel and transferred to nitrocellulose (BioRad). Monoclonal antibodies AbMSP10-1, AbMSP10-2 and AbMSP10-3 at 1 µg/mL were used for the detection of native protein PfMSP10 in schizont parasites lysates. Proteins were detected using a horseradish peroxidase-conjugated goat anti-rabbit IgG antibody (Abcam, Cambridge, MA, USA). Blots were visualized with FAST DAB (Sigma Aldrich). All antibodies were pre-depleted against non-infected red blood cells before used. Non-infected red blood cells served as a negative control. The revealed band sizes were compared using Precision Plus Protein Dual Color Standards (BioRad) and analysed in Gel Analyzer software 2010a.

The eluted fractions of purified rMSP10 were loaded onto polyacrylamide gels and analysed by western blotting, as already described, using an anti-His antibody (GenScript).

### Confocal microscopy

Smears of purified synchronized schizont stages of *P. falciparum* strain 3D7 were fixed with 100% methanol at − 20 °C for 30 min. Samples were permeabilized by treatment with 5% Triton-X-100 overnight and then rinsed with PBS three times at room temperature. Slides were blocked with 3% BSA in PBS overnight at room temperature, followed by incubation with monoclonal antibodies AbMSP10-1, AbMSP10-2, or AbMSP10-3 at 1 µg/mL, for 4 h at room temperature in darkness and then rinsed with PBS three times. AbAMA1 at 1:250 served as a control for schizont development. Then, the slides were incubated with an Alexa Fluor 488-conjugated goat anti-rabbit IgG antibody (1:1000 dilution) (Abcam) and a 1:500 diluted Alexa Fluor 594- conjugated goat anti-mouse IgG antibody (Abcam, Cambridge, MA, USA) for 1 h at room temperature in the dark and rinsed with PBS three times. The slides were mounted with VectaShield DAPI at 1 µg/mL (Vector Laboratories, Burlingame, CA, USA) mounting solution and were covered with cover slides. Parasites treated with monoclonal antibodies were visualized at 365 nm (DAPI, blue), 430 nm (AlexaFluor 488) and 504 nm (AlexaFluor 504) under a confocal microscope, ZEISS LSM 880 (Carl Zeiss, Oberkochen, Germany) at 63× magnification, zoom level 4. Non-infected red blood cells served as a negative control.

### Invasion inhibition assays (IIAs)

Synchronized schizont stages of strain 3D7 were isolated in 40%/70%/90% Percoll gradients as previously described [21]. Cultures for IIAs were established using a starting parasitaemia of 1%, at 5% haematocrit in a 100 µL final volume per well. AbMSP10-1 and AbMSP10-2 were evaluated in a range of the physiological concentrations of 10 and 100 µg/mL [22]. Mouse pre-immune sera (GenScript Laboratories) were used as negative control. All IIAs were conducted after 16 h by enumeration of infected erythrocytes on Giemsa-stained smears. Parasitaemia in treated wells was compared with parasitaemias in positive control wells, where parasites were not treated with the antibody (representing 100% invasion parasitaemia). IIAs using AbAMA1 at concentrations of 10, 50 and 100 µg/mL were performed in parallel for comparison. AbMSP10-2 served as an Ab representative of the central region of PfMSP10, for comparison with AbMSP10-1 from the N-terminal region.

### Statistical analysis

ELISA: Each patient’s serum was analysed in triplicate. Seroreactive sera were those that yielded an OD value greater than the average plus 3 standard deviations (SD) of the reactivity obtained in the serum samples from healthy donors, which were considered negative control; this value was called the cut-off. The Mann–Whitney test was used to evaluate the differences in the OD values among the groups. Fisher’s exact test (2 × 2 tables) was performed to evaluate the differences in the groups’ seroreactivity ratios. Data with a *p* value < 0.05 were considered statistically significant. The statistical analysis and the OD levels of the different samples were plotted in the GraphPad Prism software (version 3.0, GraphPad Prism, San Diego, CA, USA). IIA: All performed tests, involved at least three biological replicates and the representative results were used for analysis.

## Results

### Expression of rMSP10

**Fig. 1:**
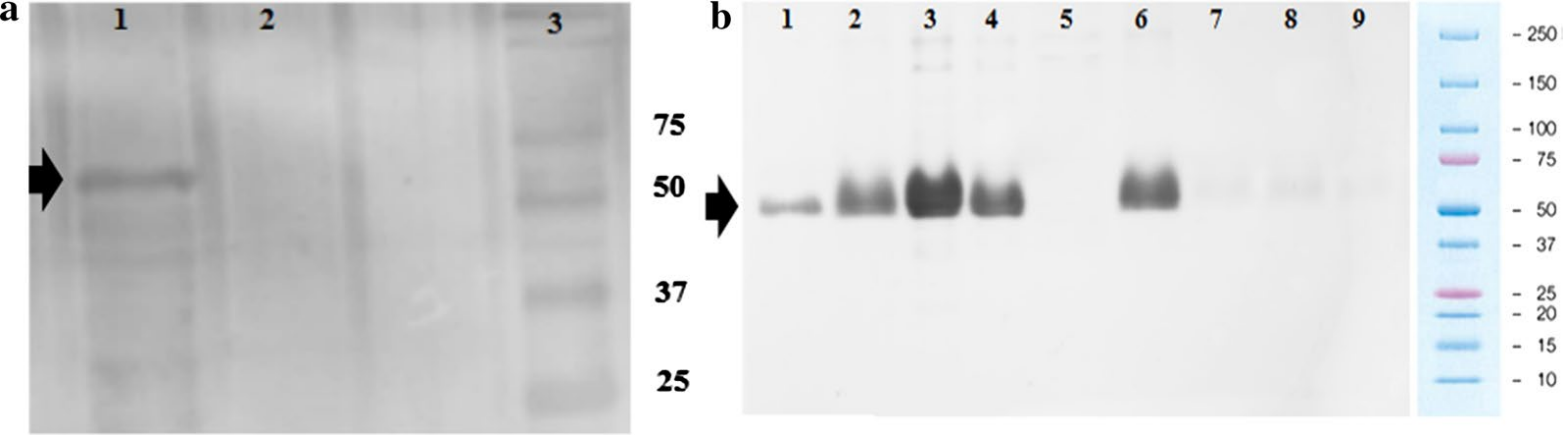
The rMSP10 western blot. ** a** *P. falciparum* Merozoite Surface Protein 10 (rMSP10) expressed in baculovirus: Lane 1: an Sf9 cell lysate from the recombinant baculovirus with the optimized *pfmsp10* gene. Lane 2: The lysate of non-infected Sf9 cells. Lane 3: molecular weight protein marker (kDa). **b** rMSP10 purified: Eluted samples obtained from the purification process with imidazole: Lane 1: 50 mM; lane 2: 150 mM; lane 3: 200 mM; lane 4: 250 mM; lane 5: lysates from uninfected Sf9 cells; lane 6: lysates from Sf9 cells infected with the recombinant baculovirus with *pfmsp10* gene; lanes 7–9: washes. Black arrows indicate rMSP10 (~ 56 kDa)

Western blot assays using the anti-His revealed the presence of a band containing a ~ 56 kDa protein, corresponding to the calculated molecular weight of rMSP10 in the Sf9-insect cell cultures infected with recombinant baculovirus P2 (Fig. 1a). Purified rMSP10 and eluted fractions were analysed by western blotting (Fig. 1b). The rMSP10 obtained was used for ELISA.

### Peptides

**Fig. 2:**
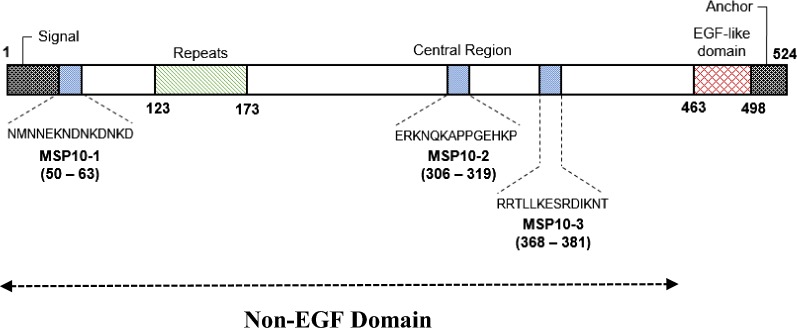
Location and sequences of PfMSP10 peptides used for this study. Positions of amino acid sequences of the peptides used for this study are revealed in a diagram of PfMSP10 protein

The three peptides selected by the bioinformatics tool (PfMSP10-1, PfMSP10-2, PfMSP10-3) corresponded to non-EGF domains of PfMSP10. Sequences from the selected peptides were different from each other and were not present in the *P. vivax* MSP10 protein sequence. PfMSP10-1 is located on N-terminal region, and both PfMSP10-2 and PfMSP10-3 are located in the central region of the protein between the repeat region and the EGF domain; as illustrated in Fig. 2.

### ELISA

**Fig. 3:**
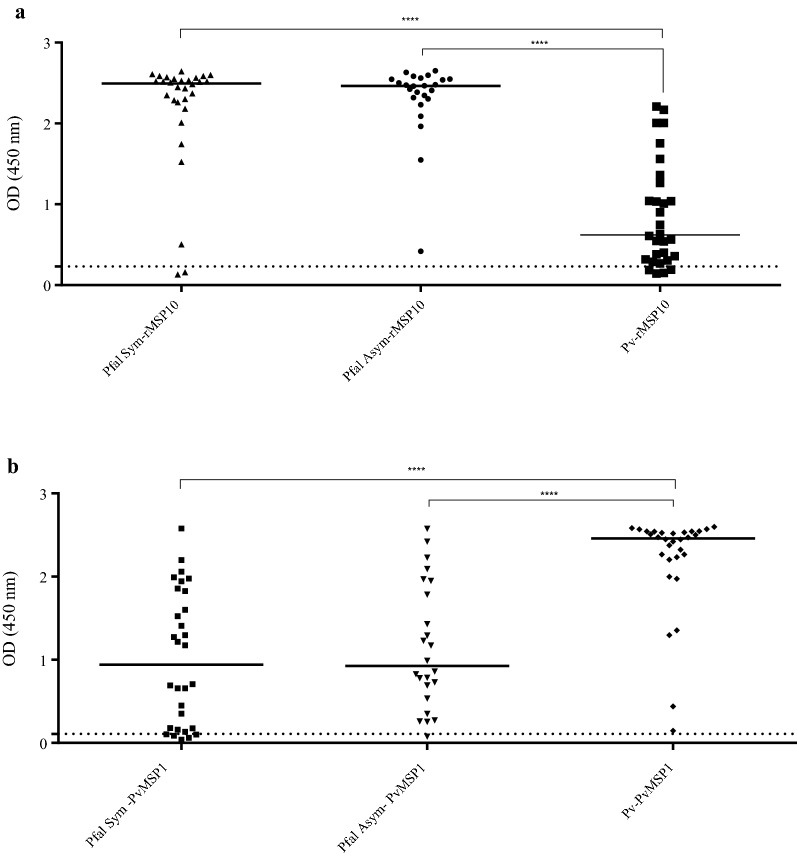
Total IgG levels from *Plasmodium falciparum* and *Plasmodium vivax*-infected individuals against antigens rMSP10 and PvMSP1. Total IgG levels in Symptomatic (Pfal Sym) and Asymptomatic (Pfal Asym) individuals infected with *P. falciparum* as well as in the *P. vivax* group (Pv) against the rMSP10 (**a**) and recombinant protein PvMSP1 (**b**), serving as a control. The dotted horizontal line indicates the calculated cut-off. The horizontal bar at the center of each group denotes the median of the group. The horizontal line for each group shows the median. The statistical significance of a difference between groups is indicated with upper horizontal branches. ***p < 0.05; ****p < 0.0001

These assays were performed to measure IgG levels using the baculovirus-expressed recombinant protein (rMSP10), and three synthetic peptides PfMSP10-1, PfMSP10-2 and PfMSP10-3. Two groups of serum samples were evaluated: the first group consisted of *P. falciparum* Sym (n = 30) and Asym (n = 24)-infected individuals and the second group was composed of *P. vivax*-infected individuals (n = 30). Serum samples from Sym and Asym *P. falciparum*-infected individuals showed a significantly higher seroreactivity with rMSP10 in contrast to the patients infected with *P. vivax* (Fig. 3a and Table 2). Interestingly, 86.7% of the individuals infected with *P. vivax* showed high seroreactivity to rMSP10 (Fig. 3a and Table 2), this result may be explained by the high identity reported between proteins PfMSP10 and PvMSP10 in the C-terminal region where two EGF domains are found (Additional file 1: Figure S4). Additionally, IgG levels during the response to the PvMSP1 antigen were evaluated in all serum samples. As expected, all the serum samples in the *P. vivax* infection group were seroreactive (100%); however; it was identified high proportion of serum samples from the *P. falciparum* group (88.8%) was seroreactive with PvMSP1 as well (Fig. 3b and Table 2). Unfortunately, one limitation of this study was the lack of information about the previous history of *P. falciparum* infection for all samples that were included in this study.

**Table 2:** Seroreactivity in *Plasmodium falciparum* Sym and Asym and *Plasmodium vivax*-infected individuals against the evaluated antigens

			Antigens		
Sera	PfMSP10-1	PfMSP10-2	− 1 + − 2	rMSP10	PvMSP1
*P. falciparum*					
Sym	4/30 (13.3%)	13/30 (43.3%)	15/30 (50.0%)	28/30 (93.3%)	25/30 (83.3%)
Asym	2/24 (4.2%)	14/24 (58.3%)	11/24 (45.8%)	24/24 (100%)	23/24 (95.8%)
*p*(Fisher’s test)	ns	ns	ns	ns	ns
*P. falciparum* Sym + Asym	6/54 (11.1%)	27/54 (50%)^a^	26/54 (48.1%)^b^	52/54 (96.3%)	48/54 (88.8%)
*P. vivax*	4/30 (8.3%)	2/30 (6.7%)^a^	1/30 (3.3%)^b^	26/30 (86.7%)	30/30 (100%)
Cut-off	0.60	0.53	0.40	0.23	0.11
*p*(Fisher test)	ns	0.0001	0.0001	ns	ns

Values denote the number of samples that responded positively to the antigen. In parenthesis, the same values are expressed in percentages

Values with the same superscript letter in the same column are statistically significantly different

*ns* columns are not statistically significantly different

**Fig. 4:**
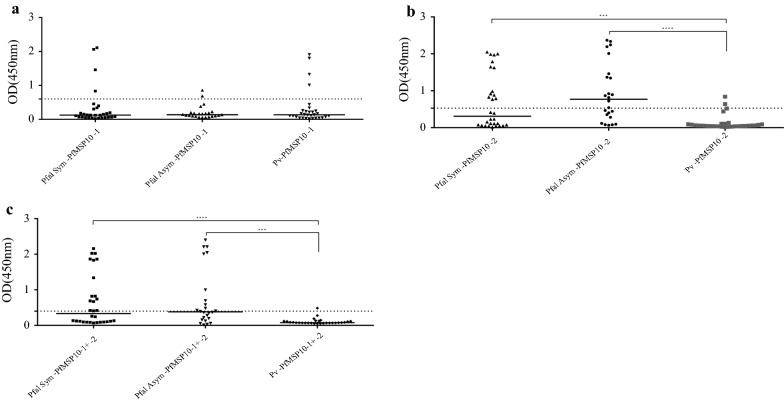
Total IgG levels from *Plasmodium falciparum* and *Plasmodium vivax*-infected individuals against PfMSP10 peptides. Total IgG levels in Symptomatic (Pfal sym) and Asymptomatic (Pfal asym) individuals infected with *P. falciparum* as well as in the *P. vivax* group (Pv) against peptides PfMSP10-1 (**a**), PfMSP10-2 (**b**) and PfMSP10-1 + -2 (**c**). The dotted horizontal line indicates the calculated cut-off. The horizontal bar at the centre of each group denotes the median of the group. The statistical significance of differences between groups is indicated with upper horizontal branches. ***p < 0.05; ****p < 0.0001

Seroreactivity with PfMSP10-1 peptide was low across all the tested groups (Fig. 4a and Table 2). The PfMSP10-2 peptide showed a significantly higher reactivity with the serum samples from groups Sym and Asym *P. falciparum*, in comparison with serum samples from *P. vivax* infected individuals (Fig. 4b and Table 2). The PfMSP10-3 peptide was also tested but showed high reactivity with healthy donors’ serum (Additional file 1: Figure S5); therefore, it was no longer used for ELISA. Interestingly, high seroreactiviy was detected in groups Sym and Asym *P. falciparum* individuals using a mixture of PfMSP10-1 + PfMSP10-2 peptides (Fig. 4c and Table 2).

Next, rMSP10 cross-reaction with other serum samples from non-*Plasmodium *spp.-infected patients’ sera was evaluated. No reaction was obtained for toxoplasma, neurocysticercosis and dengue sera (only one out of three serum samples was reactive). However, a reaction was observed for serum samples associated with leishmaniasis and leptospirosis (Additional file 1: Figure S6). No seroreaction was observed when rMSP10, PfMSP10-1 and PfMSP10-2 antigens were tested on healthy donor serum samples from endemic and non-endemic area (Additional file 1: Figure S5).

After the serological evaluation, the three synthetic peptides were employed to produce monoclonal antibodies. The monoclonal antibodies were evaluated by western blotting and confocal microscopy for the identification of native PfMSP10 in *P. falciparum* cultures, and for their functional evaluation in IIAs. All these assays allowed this study to explore the ability of the three synthetic peptides to produce functional antibodies.

### Evaluation of AbMSP10s by western blotting and confocal microscopy

**Fig. 5:**
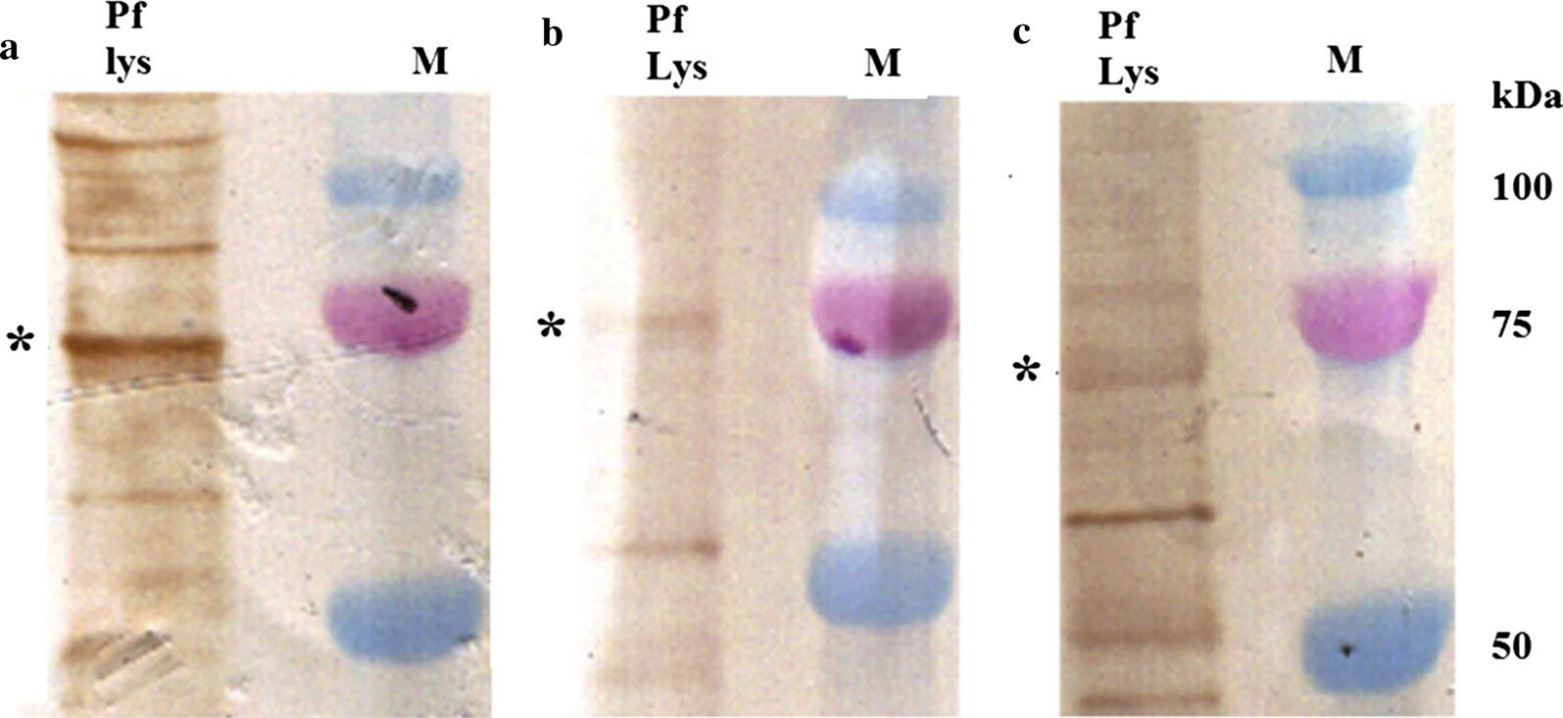
Evaluation of AbMSP10 by western blotting. AbMSP10-1 (**a**), AbMSP10-2 (**b**) and AbMSP10-3 (**c**) were used at 1 µg/mL in *P. falciparum* 3D7 schizont lysate (Pf lys), in western blot assays. Asterisks indicate the ~ 75 kDa reactive band corresponding to native PfMSP10 of *P. falciparum*. M: Protein molecular weight marker

**Fig. 6:**
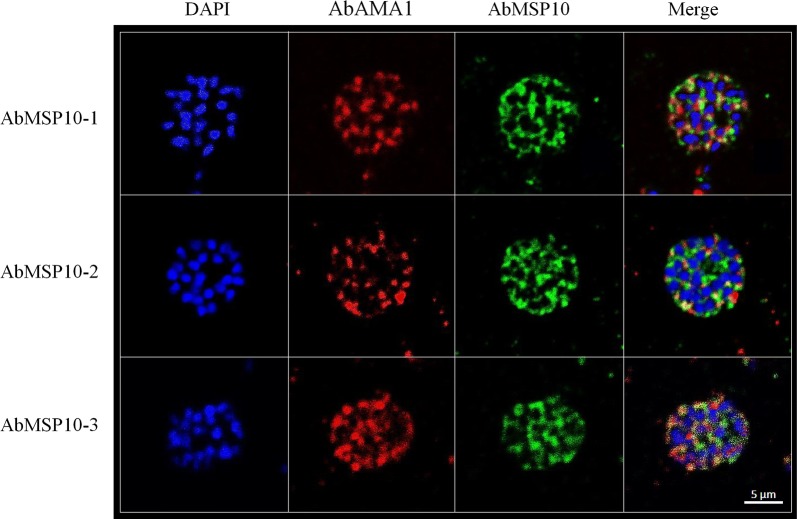
Evaluation of AbMSP10 by confocal microscopy. Purified schizont smears were fixed onto microscopy slides and incubated with antibodies AbMSP10-1, AbMSP10-2 or AbMSP10-3 at a concentration of 1 µg/mL. A strong fluorescence signal was observed for antibodies AbMSP10-1 and AbMSP10-2. AbMSP10-3 showed a dot pattern noisy signal probably due to the reduced specificity of this antibody. AbMSP10 (green), AbAMA1 (red), the nucleus of merozoites are blue (DAPI)

The western blot assay of AbMSP10-1, AbMSP10-2 and AbMSP10-3, against schizont lysates detected a ~ 75 kDa band, corresponding to the molecular weight of the PfMSP10 native protein, as already reported (Fig. 5). AbMSP10-1, AbMSP10-2 and AbMSP10-3 were evaluated by confocal microscopy in order to test whether these antibodies were capable of detecting the PfMSP10 protein on the surface of the merozoites. The results showed a strong fluorescence signal (surrounding the membrane of the merozoites) for antibodies AbMSP10-1 and AbMSP10-2, in contrast with the apical signal showed by AbAMA1 used as a control for this assay. To generate images using AbMSP10-3, the merged image showed a dot pattern noisy signal probably due to the reduced specificity of this antibody, compared to AbMSP10-1 and AbMSP10-2 (Fig. 6).

### Invasion inhibition assays (IIAs)

These assays were performed using the standard *P. falciparum* 3D7 strain to test the inhibition capabilities of AbMSP10-1 and AbMSP10-2. AbAMA1 was used, as a control, at concentrations of 10, 50 and 100 μg/mL. IIAs with AbAMA1 showed inhibition rates of 15.7, 31.2 and 45.2%, respectively. The results of testing AbMSP10-1 (N-terminal region) and AbMSP10-2 (central region), showed a lower percentage of inhibition in comparison with AbAMA1. Percentages of inhibition by AbMSP10-1 and AbMSP10-2 ranged from 10% (at a concentration of 10 µg/mL) to 24–29% at 100 µg/mL (concentration of 50 µg/mL was not determined). No statistical significant difference was observed when a comparison was made in the percentages of inhibition between AbMSP10-1 and AbMSP10-2 (Table 3).

**Table 3:** Results of inhibition of invasion assays for *Plasmodium falciparum* strain 3D7 against to monoclonal antibodies

Monoclonal antibody	Parasitaemia (%)		Inhibition of invasion (%)		
	Wild type	Pre-immune Sera	10 µg/mL	50 µg/mL	100 µg/mL
AbAMA1	6.3 ± 1.14	ND	15.7 ± 11.5	31.2 ± 9.5	45.2 ± 5.9
AbMSP10-1	8.9 ± 0.4	10.4 ± 1.0	10.1 ± 12.2	ND	29.1 ± 10.1
AbMSP10-2	4.7 ± 0.37	4.9 ± 0.7	11.8 ± 11.1	ND	24.1 ± 15.7

Each value represents an arithmetician mean and standard deviations for three biological replicates

*ND* not done

## Discussion

It was showed the potential application (to the malaria research field) of a new *P. falciparum* recombinant protein rMSP10, produced in baculovirus. Moreover, three novel peptides specific for *P. falciparum* located in the non-EGF domains of PfMSP10 and their respective monoclonal antibodies were produced and evaluated. These results have shown high seroreactivity in Sym and Asym individuals against rMSP10 corroborating the results obtained by Torres et al., that used a protein array platform [6]. In contrast, two studies carried out in the Peruvian Amazon have revealed a differential response against protein PfMSP1 (a protein with PfMSP10-like regions) between Sym and Asym *P. falciparum*-infected individuals [6, 7]. These results emphasize that PfMSP10 may not play a role of a differential immunological antigen between *P. falciparum* Sym and Asym individuals in the Peruvian Amazon region. Rather, it is proposed as a potential serological marker of exposure to falciparum malaria in this region [6].

On the other hand, PfMSP10-1 (Asn50–Asp63) peptide located at the N-terminal of PfMSP10, close to the signal peptide (Additional file 1: Figure S4) showed low seroreactivity between groups of *P. falciparum*- and *P. vivax*-infected individuals. A study reported by Lyon et al. [23], demonstrated that sera, from a monkey and a human patient both with low level of exposure to the *P. falciparum* parasite, did not react against a fragment of PfMSP1 protein (35–141aa) from the N terminal.

It has also been shown that MSP10 protein may undergo processing in a similar way to MSP1 and MSP8, but with fewer cleavage events; MSP10 final proteins (80 and 36 kDa), are C-terminal processing products of the initial full-length protein, cleaved between residues 250 and 320, at schizogony stage [9, 10]. With this, the results in this study on PfMSP10-1 point out the N-terminal region as less antigenic, some explanations for this could be the polymorphism, structure and short exposure period due to cleavage of this region [10, 24, 25]. Further functional studies can be carry out to define the role of the different regions of this protein.

PfMSP10-2 peptide (Glu306-Pro319) located within the central region and near to a potential cleavage zone of the PfMSP10 protein (Asp250-Lys320) (Additional file 1: Figure S4) [9]. High seroreactivity for this peptide was found to be similar between the Sym and Asym *P. falciparum* groups. A difference was found when *P. falciparum* groups (Sym and Asym) were compared with *P. vivax*-infected individuals (*p * <  0.0001), as explained by the fact of the uniqueness of the sequence in *P. falciparum.* The results could be suggesting the possibility of exposure of this cleavage region to the host immune system; however, a more extensive investigation is necessary to clarify the importance of this region for PfMSP10.

Studies on linear peptides from *P. falciparum* proteins (for determining a previous infection) have been reported, e.g., about the NANP peptide from of the circumsporozoite protein [12]. The present study confirmed that there are linear epitopes that are part of the antigenic repertoire of *P. falciparum*.

The results of this study suggest that PfMSP10-2 linear peptide is a *P. falciparum*-specific antigen and the potential application of this peptide in the malaria research field remains to be investigated. However, the identification of linear peptides predicted by bioinformatic tools haven contributed to the development of rapid diagnostic devices [26] and PfMSP10-2 could be a good candidate for new diagnostic and/or epidemiological tools for two reasons: (a) this peptide has good reactivity to *P. falciparum* serum samples from Peru in contrast to other recombinant proteins tested in the Amazon area (PfMSP6, PfEBA140 and GLURP) [5, 7, 27]; and, (b) in this study, PfMSP10-2 showed specificity to *P. falciparum*-infected serum samples in contrast to rMSP10, which showed cross reactivity with *P. vivax* serum samples. This phenomenon may be explained by the conserved protein domains in the *P. falciparum* species.

Elevated total IgG levels against rMSP10 was observed in sera from individuals infected with *P. falciparum* (Sym and Asym) in comparison with those infected with *P. vivax* was observed (Fig. 4). This result may be due to different sequences between PfMSP10 and PvMSP10 protein as well as the adequate folding and post-translational modifications capable of being generated using the baculovirus protein expression system [28]. It has been reported that the use of different antigenic sources (prokaryotes or eukaryotes) may have an impact on the presentation of conformational epitopes or post-translational modifications that influence its recognition [28–30]. Although, rMSP10 was obtained via a heterologous expression system in insect cells-Sf9, Black et al. expressed the carboxyl terminal region of PfMSP10 in *Escherichia coli*. However, a comparison study using 19 kDa-PvMSP1 region (from prokaryotic and eukaryotic sources) and serum samples from Brazilian patients, found no differences in seroreactivity, using antigens from both sources [31]. It is also important to consider the feasibility of large-scale production, offered by eukaryotic systems (insects, yeast and mammalian cells) in comparison to the prokaryotic ones.

It was also evaluated another point of comparison when high levels of seroreactivity to rMSP10 in sera of *P. falciparum*-infected individuals were found in contrast to the linear epitopes used in this study (Table 2). These levels may be due to the high concentration of antibodies in the serum samples that recognize conformational epitopes more than linear ones. These conformational epitopes may occur due to the presence of the EGF domains and the di-sulfide bonds that are part of these domains [9].

Serum samples from individuals infected with *P. vivax* were seroreactive with rMSP10. In the light of these results, when PfMSP10 and PvMSP10 sequences were aligned, a homologous region corresponding to the EGF domains and the GPI insertion area was noted (Additional file 1: Figure S4). It has been reported that the EGF domains are conserved among the *Plasmodium* species [9, 10]. The cross-reaction of the rMSP10 protein, with the serum samples from individuals infected with *P. vivax*, can be explained in part by the presence of circulating antibodies resulting from previous exposure to *P. falciparum*, taking into account that circulating antibodies in the Peruvian Amazon region can be detectable for up to 18 months after-*P. falciparum* infection [32, 33]. Unfortunately the limitation of this study is the lack of information about previous *P. falciparum* infection.

*Plasmodium falciparum* sub-microscopic infections (presenting as co-infections) may also explain this cross-reaction [17]. Cross-reactivity has occurred in regions similar to the Peruvian Amazon area, e.g., in China-Myanmar in experiments with PfMSP1 [34] and PfMSP5 in Indonesia [35]. Nagao et al. [36], reported results of growth inhibition of *P. falciparum* cultures by serum samples from patients experimentally infected with *P. vivax*, indicating that this phenomenon may occur in regions where the two species coexist. Thus, antibody titres against proteins shared by *P. vivax* and *P. falciparum* could be present in individuals living in endemic regions. Nonetheless, further studies are required to verify this.

Additionally, these results revealed that rMSP10 cross-react with leishmaniasis and leptospirosis positive serum samples. These findings are unexplained because there were no common sequences when BLAST searches were performed (Additional file 1: Figure S7), however the possibility of a conformational epitope cross-reaction may explain these results and the previous infections with *P. falciparum* or *P. vivax* may be taken into account.

Evaluation of rMSP10 and the PfMSP10-2 peptide as serological tools should be conducted in young individuals, travellers and experimentally exposed patients, to rule out possible reactivity factors related to the natural context before continuing with their validation in the field and regarding their cross-reactivity as an inclusion criterion for the monitoring [37]. Follow-up studies to determine the kinetics and persistence of the antibody against rMSP10 in the individuals naturally infected with *P. falciparum* in Peru should be carried out too.

It would be interesting to evaluate proteins PvMSP10 and PfMSP10 in competitive ELISAs to prove the existence of cross-reactive antibodies for both proteins. Actually, there are many studies focused in the validation of new markers, as current candidates for global sero-surveillance, as is the case for PfMSP10, PfCSP, PfMSP1-19 kDa, PfMSP3, and PfAMA-1; it should be noted that these proteins have homology with theirs *P. vivax* proteins of 58, 51, 45, 25, and 21%, respectively [3, 4].

Furthermore, in this study, novel antibodies were raised against different regions of PfMSP10, and were characterized by western blotting and confocal microscopy, to later evaluate their suitability for blocking the in vitro invasion the erythrocyte by the merozoite of *P. falciparum* strain 3D7. Results obtained by western blotting demonstrated that antibodies AbMSP10-1, AbMSP10-2 and AbMSP10-3, detected a ~ 75 kDa band in schizont lysates, which correspond to the molecular weight of the PfMSP10 native protein. These results are in agreement with those reported by Black et al. [9]. Although, seroreactivity against only one peptide (PfMSP10-2) was identified in this study. This result could be applied to grade the exposure of these epitopes in the PfMSP10 native protein during infection by *P. falciparum.*

The results using monoclonal antibodies, against the N-terminal region (AbMSP10-1) and central region (AbMSP10-2), uncovered a small percentage of inhibition, up to 29%. These data confirmed the potential role of these regions in the erythrocyte invasion. However, more studies are necessary to confirm the true role of non-EGF domains of this protein during the merozoite invasion process. It would be interesting to test whether these antibodies can also block *P. vivax* merozoite invasion of a reticulocyte in ex vivo invasion assays.

## Conclusion

PfMSP10 could be considered as a serological marker in the Peruvian Amazon Region owing to its high reactivity (rMSP10) and specificity (PfMSP10-2) in *P. falciparum*-infected individuals evaluated in this study. Even though, this protein has not been extensively investigated its applications as a potential serological marker of exposure for *P. falciparum* infections and the role of its non-EGF domains during the merozoite invasion needs further research.

## Supplementary information

**Additional file 1: Figure S1.** The Codon–optimized *pfmsp10* gene (79–1518 nu). **Figure S2**. Construction of the pFastBac-*pfmsp10* vector with insertions of codon-optimized *pfmsp10* gene (from pUC57 vector). **Figure S3.** Standard-curve of *P. falciparum* serum pool using either: a) rMSP10, b) PfMSP10-1 or b) PfMSP10-2 as antigen in ELISA assays. **Figure S4.** Amino acid sequence alignment between PfMSP10 and PvMSP10 proteins. **Figure S5.** rMSP10 cross-reaction against healthy donor sera. **Figure S6.** rMSP10 cross-reaction against other non-*Plasmodium* spp. infection sera and healthy donor sera. **Figure S7.** BLASTp results using PfMSP10 aminoacide sequence against to Leishmania (taxid:5658) or Leptospira (taxid:37387) protein data base. **Table S1**. List of antigenic peptides selected by Optimum Antigen using the amino acid sequence of PfMSP10 (GenBank:XP_966190.1).

## Data Availability

All data generated or analysed during this study are included in this published article.

## Abbreviations

rMSP10: recombinant *P. falciparum* merozoite surface protein 10
IIA: inhibition of invasion assay,
Sym: *P. falciparum* infection symptomatic
Asym: *P. falciparum* infection asymptomatic
IgG: immunoglobulin G
Ab: monoclonal antibody
AbAMA-1: *P. falciparum* apical membrane antigen 1 monoclonal antibody
EGF: epidermal growth factor
ELISA: enzyme-linked immunosorbent assay
PlasmoDB: Plasmo Genomic Data Bank
MOI: multiplicity of Infection
PMSF: phenylmethylsulfonyl fluoride
IMAC: immobilized metal affinity chromatography
MWCO: molecular weight cutoff
Pv: serum from individuals infected with *P. vivax*
PCR: polymerase chain reaction
Tx: toxoplasmosis
Ls: leishmaniasis
Lp: leptospirosis
Dn: dengue
Nc: neurocysticercosis
HDend: samples of individuals living in endemic areas with negative diagnosis for malaria by PCR and microscopy
PBS: phosphate-buffered saline
BSA: bovine serum albumin
PvMSP1: *P. vivax* merozoite surface protein 1
SDS-PAGE: sodium dodecyl sulfate polyacrylamide gel electrophoresis
DAPI: 4′,6-diamidino-2-phenylindole
OD: optical density
SD: standard deviation
