# Supplementary material for: Evaluation of Plasmodium falciparum MSP10 and its development as a serological tool for the Peruvian Amazon region
Source: Malar J. 2019 Sep 23;18:327. doi: 10.1186/s12936-019-2959-8 (PMC6757379; doi:10.1186/s12936-019-2959-8)
Supplement: Supplementary file 1 — Additional file 1: Figure S1. The Codon–optimized pfmsp10 gene (79–1518 nu). Figure S2. Construction of the pFastBac-pfmsp10 vector with insertions of codon-optimized pfmsp10 gene (from pUC57 vector). Figure S3. Standard-curve of P. falciparum serum pool using either: a) rMSP10, b) PfMSP10-1 or b) PfMSP10-2 as antigen in ELISA assays. Figure S4. Amino acid sequence alignment between PfMSP10 and PvMSP10 proteins. Figure S5. rMSP10 cross-reaction against healthy donor sera. Figure S6. rMSP10 cross-reaction against other non-Plasmodium spp. infection sera and healthy donor sera. Figure S7. BLASTp results using PfMSP10 aminoacide sequence against to Leishmania (taxid:5658) or Leptospira (taxid:37387) protein data base. Table S1. List of antigenic peptides selected by Optimum Antigen using the amino acid sequence of PfMSP10 (GenBank:XP_966190.1). [file 12936_2019_2959_MOESM1_ESM.docx]

**Additional data**

1 CACGTGGATG ACATCAAGAA CACCAGCCAA AAGAAAATTA CTTACGACAA GTACAACAAA
61 AACAAGGAGA ACATGAACAA CGAAAAGAAC GACAACAAAG ATAACAAGGA CAACATCTAC
121 AACGATAACA TTAACAACGA CAACATCAAC AACGACAACA TTAACAACGA GGACGAATAC
181 AAGTTCCTCT CAATGAAACA CTACAAGGAC TCCCTCTCTA ACAAGTTGAA CAACGAGAAC
241 GATCATATGA ACTACTTGAT CAGGAAAAGA AAGGACAACA CCCAGGGCAG TCAACACTTC
301 AACGAGAACA TCGAAAACAA CGAGAACGTC GAAAACAACG AGAACATTGA AAACAACGAG
361 AACAACGAAA ACATTGAAAA CATCGAGAAC AACGAAAACA ATGAGAACAA CGAGAACATC
421 GAGAACAACG AGAATAATGA GAATAATGAG AACTCCAGCA TCATGAACTC TGAGTCATAC
481 AACAACATCA TTAACTCCAA CGAACACAAC GAGGAACAAA TCAAGAAAAA GGAGGAAGAC
541 CTCATCGAGG CCTTCTTCCC ATTCATTTTG AAAAAGCTGG ACAACGAAAG TCTGTCGCTC
601 GATAACAAGT ACGACGATTA CTACAACCTG CCTAACGATC ACAACGACAC CCATAAGGAG
661 AACTCTTCAG ATCATAACCT GCTCGGATAC AAGCTGGGTA ACAACCTCAA ATCATACTTG
721 ATCGAGGAAA ACGACGTCAG TCAGAAAAAG ACTGACGATA TTAACGAGTC GGCTAGTTCG
781 GATTCCGAGA ACATCCAAGA AATTTTGTCC ACAGACAGCA ACACGTCTCA CCTGAAAGAA
841 AGGAAGAACC AGAAAGCTCC TCCCGGAGAG CATAAGCCTG AAGTCAAAAA CGCATTGCTG
901 AACTCACAGG TTGCGAGTCC CAAGGGAGAG GACGAGAAGA AGTCCCAGCC ACAACACCCG
961 CTGGTGAACT CCGGCGACCA GCTCCAACAT CCCAAGGAAA TCGATGAGAA CGCAGAAAAG
1021 ATTCGCCGTA CACTCTTGAA GGAGTCCCGC GACATCAAAA ACACCACTGC CATCATTGAT
1081 GAGACAGTCT ACAAGTTCGA ACAACTGATC ATGAAAGGAA GGTACTACGC TACGGCCGTG
1141 AGAAACTTCG TCATCTTCAA GGTTAACTAC ATTTGCGAGT ACAGCAAGTG TGGTCCAAAC
1201 TCTCGTTGCT ACATCGTTGA GAAAGATAAG GAACAGTGCC GCTGTCGTCC AAACTACATC
1261 GTGGACATGA GCGTCAACTA CTTCAAGTGC ATTCCGATGA AAGATATGAA CTGTTCTAAG
1321 AACAACGGTG GCTGCGACGT GAACGCTGAG TGTACGATCG TTGAAGGCGC CGTGAAGTGC
1381 CAGTGTTCCC ACCTGTACTT CGGAGACGGT GTCTTCTGTG TCAAAAACTC TCAAACCAAG
1441 CATCATCATC ATCATCATCA TCAC

**Figure S1. Codon–optimized *pfmsp10* gene (79 – 1518 nu).** Letter underline shows the 8-his tag at carboxyl side protein. The all sequence was synthetized into pUC57 vector.

pFastBacTM dual vector

PfMSP10 optimized-pUC57 vector

F1

*pfmsp10 optimized*

R1

EcoRI

BamHI

F1

Gene cloning by PCR

EcoRI

BamHI

Polh

SV40

8-His

*pfmsp10 “Optimized”* (1440 pb)

~ 6750 pb

pFastBac-*pfmsp10* vector vector

**Figure S2. Construction of the pFastBac-*pfmsp10* vector with insertions of codon-optimized *pfmsp10* gene (from pUC57 vector)**. 8-His: histidine tag; SV40: SV40 polyadenylation terminator. BamHI and EcoRI are the restriction sites where the genes were inserted into pFastBacTM dual.

**Figure S3. Standard-curve of *P. falciparum* serum pool using either: a) rMSP10, b) PfMSP10-1 or b) PfMSP10-2 as antigen in ELISA assays.** The black arrow shows the optimal serum dilution (1/100) for all ELISA procedures. This point was considered as two down dilutions from the signal saturating (1/10). The dotted horizontal line indicates the calculated cut-off. Mean and standard deviation are showed for each dilution point.

Signal Peptide (1-26aa)

PfMSP10 MMFFKCNQVFTLVFLLLLYFNNIVYTHVDDIKNTSQKKIT--------YDKYNKNKE*NMN*

PvMSP10 MKRAKCNKSLTFTIFLLLYVNGAVHVSANELNGTDGGDVPNQKDITKDYSIFERITSEG-

* ***: :*:.::****.* *:. .:::: *. .: *. ::: ..:

PfMSP10 *NEKNDNKD*NKDNIYNDN-----------INNDNINNDNINNEDEYKFLSMKHYKDSLSNK

PvMSP10 -QSASGKDD-SPISQSNTPQGEEASDGKQGNTPLDEKNSAKDVEAHFIREGHDKVTHSDV

:. . **: . * :.* * :::.* :: * :*: * * : *:

Tandem Repetas

PfMSP10 LNNENDHMNYLIRKRKDNTQGSQHFNENIENNENVENNENIENNENNENIENIENNENNE

PvMSP10 -GTE-----------EGKATGH------------VQKNAN--------------------

.* : :: * *::* *

PfMSP10 NNENIENNENNENNENSSIMNSESYNNIINSNEHNEEQIKKKEEDLIEAFFPFILK---K

PvMSP10 -------------------LRSTSYFSTQ---------------GAVSQAYNFVQENHPQ

:.* ** . :. : *: : :

PfMSP10 LDNESLSLDNKYDDYYNLPNDHNDTHKE--NSS**DHNLLGYKLGNNLKSYLIEEN**------

PvMSP10 LDNNGANVEQ-------VARGQDDVGSTENGEGNSGGEGNPLGSDKPGGKPEDASKDTPG

***:. .::: : . ::*. . ..: * **.: . *:

PfMSP10 -------**DVSQKKT**-**DDINESASSDSENIQEILSTDSNTSHLK*ERKNQKAPP***-----***GEH***

PvMSP10 NNPEESPNRNQEKGEKEKKERGDTDNPNR-------GKTSEKADQQGGNHPNGLSPDEKN

: .*:* .: :* ..:*. * .:**. ::: : * :.

PfMSP10 ***KP*E**VKN-----ALLNSQVASPKGEDEKKSQPQHPLVNSGDQLQHPKEIDENAEKI*RRTLL*

PvMSP10 NPKTHNNHSESITNPGDVGALDGEENGEGDDQTGISPTEEHPTGDAPPSDHAEKIKNTLL

:*:.:* .:*.: .**:: :.: * : : :: .:.****:.***

EGF-Like Domain 1

PfMSP10 *KESRDIKNT*TAIIDETVYKFEQLIMKGRYYATAVRNFVIFKVNYICEYSKCGPNSRCYIV

PvMSP10 KEGIDLKETTSMIDNAVYNMEQFILKTKFYTTAIRNFVHFKVNHICEYSKCGANARCYIV

**. *:*:**::**::**::**:*:* ::*:**:**** ****:******** *:*****

EGF-Like Domain 1 EGF-Like Domain 2

PfMSP10 EKDKEQCRCRPNYIVDMSVNYFKCIPMKDMNCSKNNGGCDVNAECTIVE-GAVKCQCSHL

PvMSP10 EKDKEECRCRANYMPDDSVDYFKCIPMVEKDCSKENGNCDVNAECSIDKNKDIKCQCKFN

*****:**** **: * **:******* : :***:** *******:* : :****..

GPI

PfMSP10 YFGDGVFCVKNSQTKQTLYILFIVILLVFQNFFI

PvMSP10 YIGDGIFCVMGSQAKQSLCLLLLLLICLLHKFLF

*:***:*** **:**:* :*::::: ::::*::

**Figure S4. Amino acid sequence alignment between PfMSP10 and PvMSP10 proteins**. PfMSP10 regions are shown based in Black et al and Pacheco *et al*. The figure shows, the signal peptide, Tandem Repeats, EGF-like domains and glycosylphosphatidylinositol (GPI). Letters in italic and underlined show selected peptides (PfMSP10-1, PfMSP10-2 and PfMSP10-3). In bold, the area predicted by Black *et al* for the cleavage of the PfMSP10 protein that gives rise to a 36kDa protein.

**Figure S5. rMSP10 cross-reaction against healthy donor sera.** Total IgG levels from Symptomatic (Pfal Sym) and Asymptomatic (Pfal Asym) individuals infected with *P. falciparum*. HDend: Healthy donor from endemic area for Malaria and HDn: Healthy donor from non-endemic area. The dotted horizontal line indicates the calculated cut-off. The horizontal bar at the center of each group indicates the median of the group.

**Figure S6. rMSP10 cross-reaction against other non-*Plasmodium spp* infection sera and healthy donor sera.** Total IgG levels from Symptomatic (Pfal Sym) and Asymptomatic (Pfal Asym) individuals infected with *P. falciparum*. HDend: Healthy donor from endemic area for Malaria; Tx: Toxoplasmosis; Ls: Leishmaniasis; Lp: Leptospirosis; Nc: Neurocysticercocys and Dn: Dengue. The dotted horizontal line indicates the calculated cut-off. The horizontal bar at the center of each group indicates the median of the group.

*
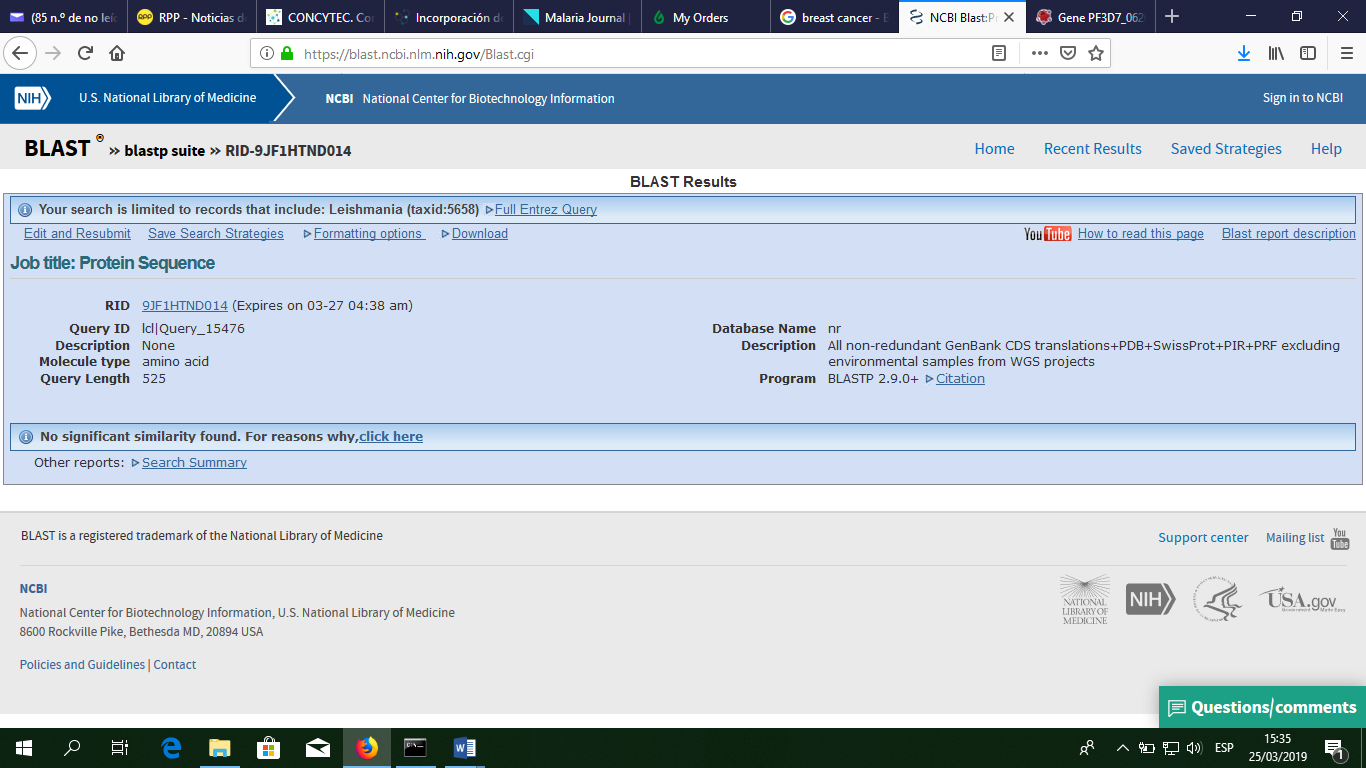
Leishmania (taxid:5658)*

*
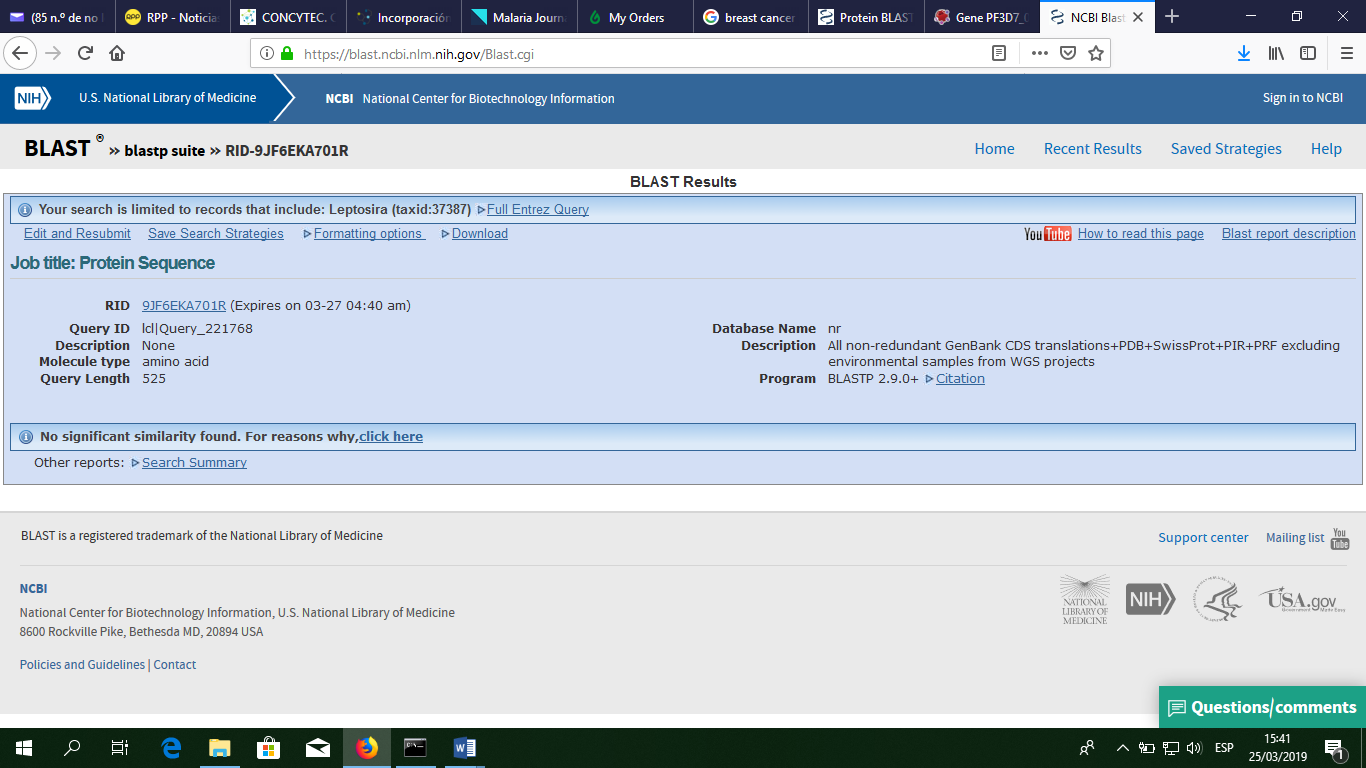
Leptosira (taxid:37387)*

**Figure S7. BLASTp results using PfMSP10 aminoacide sequence against to Leishmania (taxid:5658) or Leptosira (taxid:37387) protein data base.** The program selection/algorithm was Blastp (protein-protein BLAST). Evaluated in March,2019.

**References:**

Black CG, Wang L, Wu T, Coppel RL. Apical location of a novel EGF-like domain-containing protein of Plasmodium falciparum. Molecular and biochemical parasitology. 2003;127(1):59-68.

Pacheco MA, Elango AP, Rahman AA, Fisher D, Collins WE, Barnwell JW, et al. Evidence of purifying selection on merozoite surface protein 8 (MSP8) and 10 (MSP10) in Plasmodium spp. Infection, genetics and evolution : journal of molecular epidemiology and evolutionary genetics in infectious diseases. 2012;12(5):978-86.

Stephen F. Altschul, Thomas L. Madden, Alejandro A. Schäffer, Jinghui Zhang, Zheng Zhang, Webb Miller, and David J. Lipman (1997), "Gapped BLAST and PSI-BLAST: a new generation of protein database search programs", Nucleic Acids Res. 25:3389-3402.

| **No** | **Antigenic Determinant** | **Length** | **Antigenicity/Surface/Hydrophilicity** | **Disordered Score** | **Mus_musculus/ Oryctolagus_cuniculus blast** | **In our study** |
| --- | --- | --- | --- | --- | --- | --- |
| 1* | NMNNEKNDNKDNKD | 14 | 3.27/1.00/1.46 | 0.1597 | 64%/56% | PfMSP10-1 |
| 2* | ERKNQKAPPGEHKP | 14 | 3.16/0.93/1.14 | 0.1376 | 49%/57% | PfMSP10-2 |
| 3* | RRTLLKESRDIKNT | 14 | 2.85/0.79/0.92 | 0.1538 | 50%/49% | PfMSP10-3 |
| 4 | VASPKGEDEKKSQP | 14 | 2.55/0.93/1.26 | 0.0930 | 56%/49% | - |
| 5 | IRKRKDNTQGSQHF | 14 | 2.38/0.86/0.78 | NONE | 56%/49% | - |
| 6 | SGDQLQHPKEIDEN | 14 | 2.00/0.79/0.74 | 0.1314 | 56%/49% | - |
| 7 | NNLKSYLIEENDVS | 14 | 1.84/0.71/0.38 | NONE | 49%/56% | - |
| 8 | IINSNEHNEEQIKK | 14 | 1.65/0.79/0.85 | NONE | 56%/56% | - |
| 9 | SLSNKLNNENDHMN | 14 | 1.65/0.79/0.43 | NONE | 70%/56% | - |
| 10 | INNDNINNDNINNE | 14 | 1.55/0.86/0.52 | 0.1126 | 56%/64% | - |

**Table S1. List of antigenic peptides selected by Optimum Antigen using the amino acid sequence of PfMSP10 (GenBank:XP_966190.1).**

* indicated the peptides with higher
